# Exploring Hesperidin Hydrogel for Pulp Capping Advancements: An In Vitro Study

**DOI:** 10.7759/cureus.81168

**Published:** 2025-03-25

**Authors:** Swetha Geervani V, Kiran Kumar Neelakantappa, Manimozhi M, Abhishek M, Seema Merwade, Savitha B Naik

**Affiliations:** 1 Department of Conservative Dentistry and Endodontics, Government Dental College and Research Institute, Bengaluru, IND

**Keywords:** biodentine, hesperidin, hydrogels, pulp capping and pulpectomy, regenerative endodontics, vascular endothelial growth factor (vegf)

## Abstract

Background

Regenerative endodontics is revolutionizing pulp preservation by utilizing bioactive materials to enhance dentinogenesis. Direct pulp capping, a widely used technique, often relies on traditional pulp-capping agents (PCAs) like Biodentine. However, limitations such as reduced long-term bioactivity highlight the need for novel alternatives. Hesperidin, a natural flavonoid with potent anti-inflammatory and angiogenic properties, has emerged as a promising candidate. This study investigates and compares the growth factor release profiles of hesperidin hydrogel and Biodentine to assess their potential in vital pulp therapy.

Methods

Sixty extracted human third molars were processed into dentin discs and divided into two groups: hesperidin hydrogel and Biodentine. Each group was further subdivided based on incubation durations (7, 14, and 28 days). The release of vascular endothelial growth factor (VEGF), a key regulator of angiogenesis and tissue regeneration, was quantified using enzyme-linked immunosorbent assay (ELISA). In vitro permeation studies and scanning electron microscopy (SEM) analyses were performed to assess material properties. Statistical analysis was conducted using IBM SPSS Statistics for Windows, Version 26.0 (Released 2019; IBM Corp., Armonk, NY, United States).

Results

Hesperidin hydrogel demonstrated a significantly higher and sustained release of VEGF compared to Biodentine across all time intervals (p < 0.0001). While Biodentine showed an initial burst release that declined over time, hesperidin hydrogel maintained stable bioactivity, indicating prolonged regenerative potential.

Conclusion

Hesperidin hydrogel outperforms Biodentine in sustaining VEGF release, making it a promising next-generation biomaterial for direct pulp capping. Its ability to promote angiogenesis and support long-term pulp vitality highlights its potential to revolutionize regenerative endodontics. Further in vivo studies are needed to confirm its clinical efficacy.

## Introduction

Dentinogenesis, the process of forming dentin, is a fundamental biological response to pulp injury and occurs through two distinct mechanisms: reactionary dentinogenesis and reparative dentinogenesis. Reactionary dentinogenesis occurs in response to mild, non-exposed pulp injuries, where pre-existing odontoblasts are stimulated to secrete new dentin as a protective barrier. In contrast, reparative dentinogenesis occurs following severe injuries with pulp exposure, necessitating the recruitment of dental pulp stem cells (DPSCs) or progenitor cells, which then proliferate and differentiate into odontoblast-like cells to restore lost dentin [1,2].

While the distinction between these two processes is often based on the presence or absence of pulp exposure, their biological mechanisms are more complex, involving intricate molecular signaling pathways that regulate cell recruitment, differentiation, and matrix deposition. The wound-healing process in the pulp progresses through overlapping stages, with the successful recruitment and proliferation of progenitor cells being crucial for effective tissue regeneration [3,4].

Growth factors play a vital role in promoting vascularization, mineralization, and stem cell proliferation, thereby enhancing the repair and regeneration of the pulp-dentin complex [5]. Among them, bone morphogenetic protein-7 (BMP-7), transforming growth factor-beta 1 (TGF-β1), and vascular endothelial growth factor (VEGF) are particularly important as they contribute to cellular differentiation, angiogenesis, and extracellular matrix formation, all of which are essential for successful pulp healing [6].

Regenerative endodontic procedures (REPs) have emerged as a promising biological approach to pulp preservation and repair, aiming to restore a fully functional pulp-dentin complex using bioactive materials and signaling molecules.

Advancements in regenerative science have shifted the focus from pulpal repair to complete pulpal regeneration. Clinically, one of the most widely used strategies for pulp preservation is direct pulp capping, which involves applying a pulp-capping agent (PCA) to an exposed pulp to promote healing and maintain vitality [7]. However, despite its potential, direct pulp capping remains technically challenging, with long-term success rates as low as 9% [8], underscoring the need for more effective PCAs that enhance pulp healing and dentinogenesis.

Among the current PCAs, Biodentine, a calcium silicate-based material, has gained widespread recognition for its ability to stimulate reparative dentinogenesis by releasing TGF-β1, which facilitates cellular differentiation and tissue repair [9]. However, despite its widespread use, Biodentine has limitations, such as reduced long-term bioactivity, long-term solubility, higher heavy metal release, and dose- and time-dependent cytotoxicities [10]. A potential drawback of using Biodentine is that, although it supports pulp preservation, the overall predictability of treatment outcomes may be compromised. Traditional management of deep carious lesions requires the complete removal of infected and affected dentin, which can disrupt the dentin barrier and reduce treatment reliability. In this context, Biodentine demonstrated a pulp survival rate of 82.6% after 1-1.5 years, indicating a notable risk of treatment failure [11]. This necessitates the need for more durable and biologically active alternatives, such as hesperidin, a natural flavanone glycoside found in citrus fruits, which are being explored to improve clinical outcomes.

Hesperidin possesses anti-inflammatory, antioxidant, and immunomodulatory properties that help reduce capillary permeability, control bleeding, and minimize edema within the pulp [12]. In hydrogel formulations, hesperidin has also demonstrated wound-healing properties, making it a promising candidate for vital pulp therapy [13].

Given the growing interest in bioactive materials, comparing the growth factor release profiles of different PCAs may serve as a predictive measure of treatment success.

This study aimed to assess, evaluate, and compare the growth factor release of hesperidin hydrogel and Biodentine, contributing to the development of more effective regenerative materials for endodontic applications.

## Materials and methods

Preparation of dentin discs

Sixty extracted human third molars were collected from healthy patients after obtaining written consent. After extraction, the soft tissue around the teeth was removed using a periodontal curette (GDC Universal, Hoshiarpur, India) and washed with normal saline for five minutes. The teeth were embedded in acrylic blocks, covering the root portion. The coronal portions were horizontally sectioned using a precision cutting machine (Minitom, Struers, Champigny-sur-Marne, France) with a diamond‑edged blade at low speed (150 rpm) and continuous cooling with phosphate-buffered saline (PBS) (1X). This low-speed sectioning with constant PBS cooling prevented the denaturation of organic and protein contents in the dentin matrix. The resulting dentin discs measured 2 mm × 2 mm. The discs were then disinfected with 1.5% sodium hypochlorite (NaOCl) (Hyposol™) for five minutes to remove organic debris. Finally, the blocks were stored in sterile PBS until further surface treatment and growth factor quantification.

Preparation of hydrogel

About 1.0% grams of Carbopol was dispersed in 100 mL of water by stirring for 30 minutes using an overhead stirrer at around 500 rpm. Then, approximately 100 mg of hesperidin (Otto Chemie Pvt. Ltd., Mumbai, India) was gradually added to the vessel containing Carbopol.

**Figure 1:**
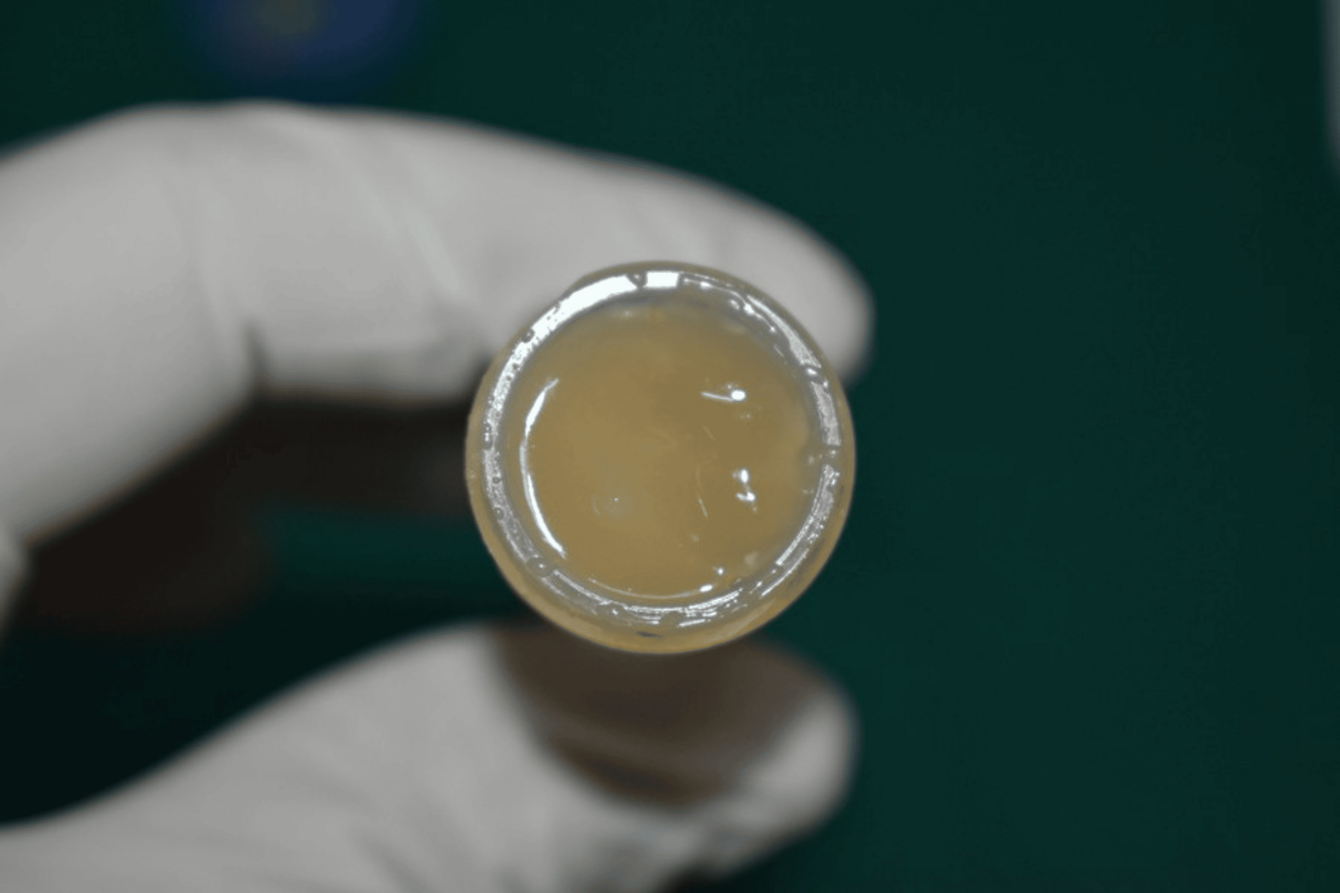
Prepared hesperidin hydrogel

To enhance the antibacterial properties of the hydrogel, 0.6 mg of eugenol per 100 g of gel was incorporated. This concentration was chosen to minimize bacterial contamination while ensuring biocompatibility [14]. The pH was adjusted to 6.7 using 0.1 N NaOH. The translucent gel was then placed in a plastic container and stored in a refrigerator. The prepared hydrogel is shown in Figure 1.

In vitro permeation studies

Permeation Procedure

In vitro permeation studies were performed in triplicate over 48 hours using a Franz diffusion cell (FDC) with an effective diffusional area of 0.79 cm^2^. The 48-hour period was chosen based on prior studies, which suggest that initial growth factor release is critical in early wound healing, influencing cellular differentiation and vascularization. The apparatus consisted of a glass receiver chamber with a sampling port on one side and two parallel tubes on the other side for water circulation to maintain a constant and desired temperature as per the experimental demand. The upper open end of the receiver was grounded to mount the dialysis or cellophane membrane (HiMedia Laboratories, Thane, India), which was clamped securely to prevent leakage between the donor and receptor compartments. A small magnetic bead was placed at the bottom of the receiver chamber to facilitate mixing during the experiment. The dialysis membrane was mounted between the receiver and donor compartment of the FDC with an area of 0.79 cm^2^. Approximately 0.5 g of hesperidin gel was dispensed into the donor compartment, while the receiver compartment was filled with PBS (pH 6.8). The system was stirred at 600 ± 10 rpm using a magnetic bead in an FDC assembly.

At predetermined time points (0, 1, 2, 3, 4, 6, 8, 10, 12, 24, 36, and 48 hours), 0.5 mL samples were withdrawn from the receptor compartment, and an equal volume was replaced with 6.8 pH buffer to maintain sink conditions. The withdrawn samples were analyzed using a UV instrument (UV-1900i, Shimadzu, Kyoto, Japan), and the results were recorded.

Scanning electron microscopy (SEM) analysis

The surface morphology of the hydrogel was studied using scanning electron microscopy (SEM; Hitachi 3400S, Hitachi, Japan).

Conditioning of dentin blocks

The obtained samples were randomly divided into six groups based on the conditioning agent used.

Group 1: Hesperidin Hydrogel Group

Group 1A: Ten dentin discs were conditioned using the prepared hydrogel. The conditioned blocks were then transferred to graduated centrifugation tubes, stored in PBS solution (0.5 mL), and incubated at 37°C (ALPHA) for seven days. The tooth slice-conditioned medium was collected for enzyme-linked immunosorbent assay (ELISA) quantification of VEGF (Elabscience, Houston, TX, United States).

Group 1B: Another 10 dentin discs were conditioned with the prepared hydrogel. The discs were then washed, transferred to graduated centrifugation tubes, and stored in PBS solution (0.5 mL) for 14 days at 37°C (ALPHA). The tooth slice-conditioned medium was collected for ELISA quantification of VEGF.

Group 1C: Ten dentin discs were conditioned using the prepared hydrogel. The conditioned discs were then transferred to graduated centrifugation tubes, stored in PBS (0.5 mL), and incubated at 37°C (ALPHA) for 28 days. Following the incubation period, tooth slice-conditioned medium was collected for ELISA quantification of VEGF.

Group 2: Biodentine Group

Group 2A: Biodentine was freshly mixed according to the manufacturer’s instructions, applied to 10 dentin discs, and then allowed to set at 37°C for four hours. The conditioned blocks were then transferred to graduated centrifugation tubes, stored in PBS solution (0.5 mL), incubated at 37°C (ALPHA) for seven days, and then processed as mentioned above.

Group 2B: Biodentine was exposed to 10 dentin discs for 14 days and stored and processed as described above.

Group 2C: Biodentine was freshly mixed according to the manufacturer’s instructions and applied to 10 dentin discs. The material was incubated at 37°C for four hours. The conditioned discs were then transferred to graduated centrifugation tubes, stored in PBS (0.5 mL), and incubated at 37°C (ALPHA) for 28 days. Following the incubation period, the samples were processed as described previously for ELISA quantification of VEGF.

Statistical analysis

Data were analyzed using IBM SPSS Statistics for Windows, Version 26.0 (Released 2019; IBM Corp., Armonk, NY, United States). The level of significance was set at p < 0.05. Descriptive statistics were used to calculate the means and standard deviations for each group, and normality was assessed with the Shapiro-Wilk test. Inferential statistics included the independent t-test for between-group differences and repeated measures ANOVA for within-group analysis, followed by the Bonferroni post hoc test.

## Results

In vitro permeation studies

**Figure 2:**
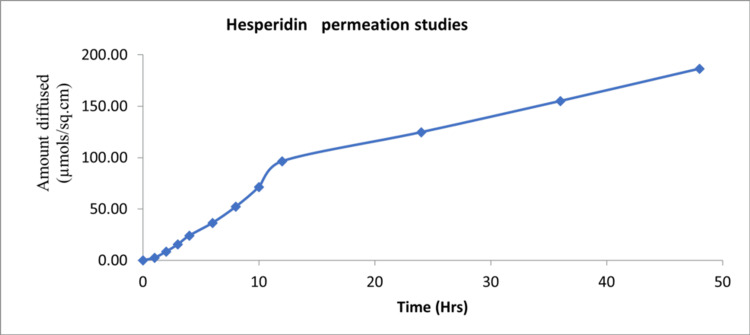
Standard curve depicting results of the 48-hour permeation test

The results showed a strong linear relationship between concentration (µg/mL) and absorbance, with an R² value near 1, indicating excellent linearity. The coefficient of variation (CV, %) was generally low (<5%), indicating good precision. The standard curve data demonstrated consistent measurements with minimal variation (Figure 2). These findings indicate sustained release of hesperidin from the hydrogel for the 48-hour period.

Scanning electron microscopy (SEM) analysis

**Figure 3:**
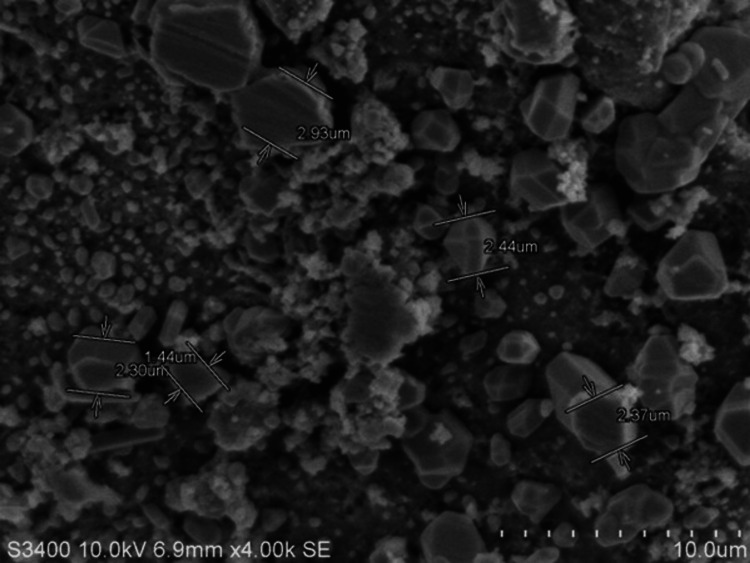
Scanning electron microscopy images of the gel surface showing hesperidin particles within the Carbopol gel matrix and eugenol White arrow marks show hesperidin particles in micrometer size.

Figure 3 shows SEM images of hesperidin particles embedded in a Carbopol gel matrix.

Comparison between Biodentine and hesperidin hydrogel

Table 1 presents the between-group comparison of Biodentine and hydrogel at 7, 14, and 28 days using an independent t-test. Significant differences were observed at all time intervals (7, 14, and 28 days) between the two treatments (p < 0.0001). The mean values for Biodentine were significantly lower than those for hydrogel, with the greatest difference observed at 7 days (968.65), followed by 14 days (1056.80), and 28 days (784.484). These results indicate that hydrogel consistently exhibited higher values than Biodentine across all time points.

**Table 1:** Between-group comparison of Biodentine and hydrogel at 7, 14, and 28 days using an independent t-test All intervals show statistically significant differences in vascular endothelial growth factor (VEGF) release.

Interval	Treatment	Mean (VEGF levels in picograms)	Std. deviation	t-value	p-value	Difference
7 days	Biodentine	552.6866	207.724	14.73	0.0001*	968.65
	Hydrogel	1521.3369	8.2418			
14 days	Biodentine	488.6776	232.404	14.37	0.0001*	1056.80
	Hydrogel	1545.4807	8.63614			
28 days	Biodentine	725.9974	203.154	14.10	0.0001*	784.484
	Hydrogel	1510.4816	134.782			

Table 2 presents post hoc comparisons for Biodentine using the Bonferroni test. No significant differences were observed between the 7-day and 14-day groups (p = 1.000) or between the 7-day and 28-day groups (p = 0.247). However, a significant difference was found between the 14-day and 28-day groups (p = 0.040), with the 28-day group showing lower values.

**Table 2:** Post hoc comparisons for Biodentine using the Bonferroni test Results show that there is a significant reduction in VEGF release after 28 days.

(I) group	(J) group	Mean difference (I-J)	Std. error	p-value	95% confidence interval	
					Lower bound	Upper bound
7 days	14 days	64.00897	96.06698	1.000	-181.1980	309.2160
	28 days	-173.3108	96.06698	0.247	-418.5178	71.8962
14 days	28 days	-237.3198	96.06698	0.040*	-482.5268	7.8872

Table 3 presents the post hoc comparison results for the VEGF release using hydrogel, analyzed with the Bonferroni test. No significant differences were observed between the 7-day and 14-day groups (p = 1.000), the 7-day and 28-day groups (p = 1.000), or the 14-day and 28-day groups (p = 0.976), as all p-values exceeded the significance threshold. These findings indicate that no statistically significant differences were found between any of the groups.

**Table 3:** Post hoc comparison of the VFGF release using hydrogel No significant differences were found, indicating sustained vascular endothelial growth factor (VEGF) release.

(I) group	(J) group	Mean difference (I-J)	Std. error	p-value	95% confidence interval	
					Lower bound	Upper bound
7 days	14 days	-24.14373	34.9368	1.000	-113.3185	65.0310
	28 days	10.85532	34.9368	1.000	-78.3194	100.0301
14 days	28 days	34.99906	34.9368	0.976	-54.1757	124.1738

**Figure 4:**
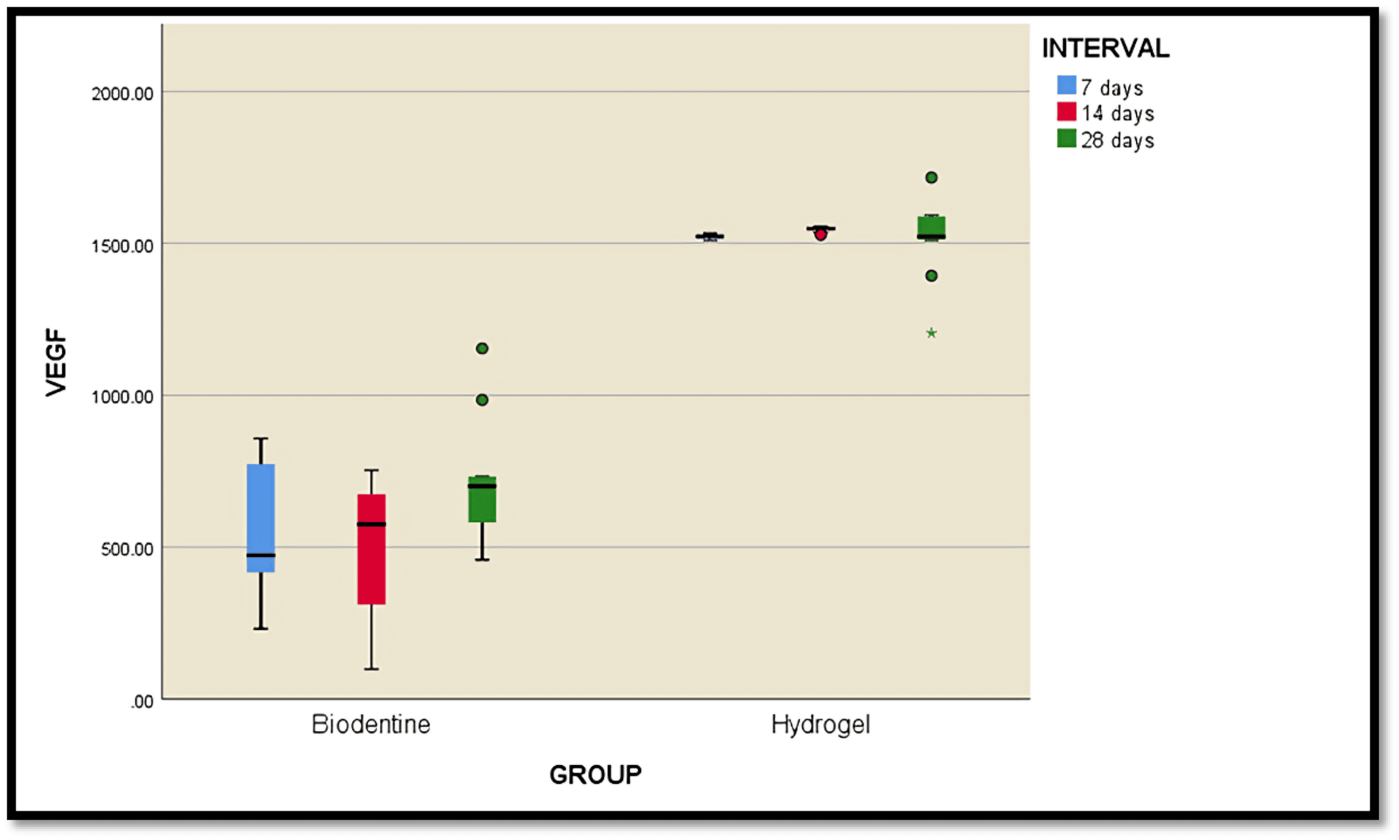
Box plot representing the overall comparison between the groups and subgroups VEGF: vascular endothelial growth factor.

The overall result is plotted in Figure 4.

## Discussion

This study aimed to evaluate the efficacy of hesperidin hydrogel as a PCA in comparison with Biodentine, with a particular focus on its ability to release VEGF. VEGF is a crucial signaling molecule in tissue regeneration, primarily facilitating angiogenesis, cell proliferation, and extracellular matrix formation, all of which are essential for pulp healing and dentinogenesis [4]. The results showed that hesperidin hydrogel exhibited a significantly higher and sustained release of VEGF than Biodentine.

The ability of hesperidin hydrogel to maintain consistent VEGF levels over time suggests that it may provide more prolonged bioactivity, which is essential for the long-term success of REPs [15].

Previous studies have established Biodentine as a widely used calcium silicate-based biomaterial capable of stimulating reparative dentinogenesis, a process that involves the deposition of mineralized tissue by odontoblast-like cells. Biodentine has been shown to induce mineralized foci within two days of application, with an increase in mineral deposition over the following weeks. Similar responses have been observed with mineral trioxide aggregate (MTA), another widely studied pulp-capping material [5]. Mineral trioxide aggregate (MTA) presents some drawbacks that may limit its clinical application. Studies have reported moderate success rates for direct pulp capping with MTA, such as 80.5% with a median follow-up of 3.5 years [16] and 80.3% in a separate study with a 1.2-year follow-up [17], indicating some degree of treatment failure over time. Additionally, one of the most significant disadvantages of MTA is its potential to cause crown discoloration. Clinical findings have shown that discoloration occurred in 60% of primary teeth treated with gray MTA [18] and in 13.6% of permanent teeth following direct pulp capping with white MTA [19]. Furthermore, MTA is also associated with a relatively long setting time, which can complicate clinical handling and delay treatment procedures. These factors collectively highlight the limitations of MTA in certain clinical scenarios.

However, despite its regenerative capabilities, Biodentine exhibited a decline in VEGF levels between the 14-day and 28-day intervals, suggesting a limited duration of bioactivity. The observed decline in VEGF levels in the Biodentine group after 14 days may be attributed to the initial burst release of calcium ions and growth factors, which diminished over time due to material degradation and reduced ion exchange capacity.

In contrast, hesperidin hydrogel maintained steady VEGF levels across all time points, indicating that it may be more effective in sustaining a regenerative microenvironment conducive to dentin formation and pulp healing.

The enhanced performance of hesperidin hydrogel can be attributed to its polyphenolic structure, which has been widely recognized for its antioxidant, anti-inflammatory, and angiogenic properties. Flavonoids, such as hesperidin, upregulate angiogenic markers, thereby facilitating neovascularization in injured tissues [20]. Additionally, hesperidin plays a role in reducing oxidative stress, which is crucial for preventing premature cell apoptosis and supporting tissue regeneration. The modulation of inflammatory pathways by hesperidin further ensures that excessive immune responses do not interfere with pulp healing or cellular differentiation [21,22]. These properties collectively contribute to its stronger and more sustained regenerative potential than traditional calcium silicate-based materials such as Biodentine. Hesperidin was previously used in a paste form in an animal study for direct pulp capping [13,23].

Another key factor in the superior performance of hesperidin hydrogel is its delivery system. The hydrogel formulation provided a controlled and prolonged release of hesperidin, ensuring that the bioactive compound remained available over an extended period. This controlled-release mechanism prevents a sudden spike in concentration, which can often lead to rapid degradation and loss of biological activity. Instead, the polymer matrix of the hydrogel likely regulates drug diffusion and degradation, allowing gradual and sustained exposure of pulp tissues to hesperidin. This slow and continuous bioavailability closely mimics the natural release of growth factors during dentinogenesis, which occurs in a regulated and prolonged manner, rather than as a short-lived pulse. Bioactive molecules have a short half-life and require high local concentrations for their effectiveness [7].

The findings of this study suggest that hesperidin hydrogels may overcome this challenge by providing a sustained and consistent bioactive effect, ensuring an optimal healing environment for extended periods.

The clinical implications of these findings suggest that hesperidin hydrogel could serve as a more effective alternative to Biodentine for vital pulp therapy. The higher levels of VEGF release observed in hesperidin hydrogels indicate that they have a stronger capacity to promote angiogenesis, which is crucial for ensuring adequate blood supply to regenerate pulp tissue [20].

The ability of hesperidin hydrogel to provide sustained bioactivity may also lead to more stable and predictable long-term outcomes in direct pulp-capping procedures. Additionally, its anti-inflammatory properties may contribute to better tissue compatibility and reduce the risk of chronic inflammation and pulp necrosis, which are common causes of treatment failure in direct pulp capping. Beyond endodontics, the regenerative properties of hesperidin hydrogel open possibilities for its use in broader dental and periodontal applications, particularly in procedures requiring enhanced soft and hard tissue healing [24].

The findings of this study revealed that hesperidin hydrogel consistently released VEGF at a significantly higher rate and for a prolonged duration compared to Biodentine across all evaluated time points (7, 14, and 28 days) [25,26], suggesting its enhanced potential as a bioactive pulp-capping material.

Despite the promising results of this study, certain limitations must be acknowledged. Since the experiments were conducted in vitro, they did not fully replicate the complex biological interactions that occur within the in vivo pulp-healing environment. Future studies involving animal models and clinical trials are necessary to validate these findings and to confirm the long-term effectiveness of hesperidin hydrogels in real-world clinical scenarios. Additionally, while this study focused primarily on VEGF, further investigations should explore the effects of hesperidin hydrogel on other key growth factors involved in pulp regeneration, such as BMP-7 and TGF-β1, to gain a comprehensive understanding of its regenerative potential. Another important consideration is the long-term stability and biodegradability of hesperidin hydrogel, as these factors will play a critical role in determining its practical feasibility for routine clinical use [27].

## Conclusions

This study provides strong evidence that hesperidin hydrogel has superior regenerative potential compared to Biodentine, particularly owing to its higher and sustained VEGF release. Its ability to promote angiogenesis, reduce inflammation, and sustain bioactivity over prolonged periods makes it a promising candidate for direct pulp-capping and other regenerative endodontic applications. Further clinical validation and optimization of its mechanical properties could establish it as a next-generation biomaterial for endodontic applications.

## References

[1] Cooper PR, Takahashi Y, Graham LW, Simon S, Imazato S, Smith AJ (2010). Inflammation-regeneration interplay in the dentine-pulp complex. J Dent.

[2] Cooper PR, Chicca IJ, Holder MJ, Milward MR (2017). Inflammation and regeneration in the dentin-pulp complex: net gain or net loss?. J Endod.

[3] Duncan HF, Cooper PR, Smith AJ (2019). Dissecting dentine-pulp injury and wound healing responses: consequences for regenerative endodontics. Int Endod J.

[4] Tomson PL, Lumley PJ, Smith AJ, Cooper PR (2017). Growth factor release from dentine matrix by pulp-capping agents promotes pulp tissue repair-associated events. Int Endod J.

[5] About I (2013). Dentin-pulp regeneration: the primordial role of the microenvironment and its modification by traumatic injuries and bioactive materials. Endodontic Topics.

[6] Gaviño-Orduña JF, Caviedes-Bucheli J, Manzanares-Céspedes MC (2021). Dentin growth after direct pulp capping with the different fractions of plasma rich in growth factors (PRGF) vs. MTA: experimental study in animal model. J Clin Med.

[7] Whitehouse LL, Thomson NH, Do T, Feichtinger GA (2021). Bioactive molecules for regenerative pulp capping. Eur Cell Mater.

[8] Bjørndal L, Fransson H, Bruun G (2017). Randomized clinical trials on deep carious lesions: 5-year follow-up. J Dent Res.

[9] Wattanapakkavong K, Srisuwan T (2019). Release of transforming growth factor beta 1 from human tooth dentin after application of either ProRoot MTA or biodentine as a coronal barrier. J Endod.

[10] Ravi H, Mahendran K, Velusamy V, Babu S (2021). Biodentine- a review on its properties and clinical applications. Journal of Biomedical and Pharmaceutical Research.

[11] Lipski M, Nowicka A, Kot K (2018). Factors affecting the outcomes of direct pulp capping using biodentine. Clin Oral Investig.

[12] Abo El-Mal EO, Abu-Seida AM, El Ashry SH (2021). Biological evaluation of hesperidin for direct pulp capping in dogs' teeth. Int J Exp Pathol.

[13] Bagher Z, Ehterami A, Safdel MH (2020). Wound healing with alginate/chitosan hydrogel containing hesperidin in rat model. J Drug Deliv Sci Technol.

[14] Kowalewska A, Majewska-Smolarek K (2023). Eugenol-based polymeric materials-antibacterial activity and applications. Antibiotics (Basel).

[15] Khan JA, Hasan A, Dossa S, Ali B (2021). Effect of natural and artificial dentin conditioners on the release of vascular endothelial growth factor. J Endod.

[16] Mente J, Hufnagel S, Leo M (2014). Treatment outcome of mineral trioxide aggregate or calcium hydroxide direct pulp capping: long-term results. J Endod.

[17] Hilton TJ, Ferracane JL, Mancl L (2013). Comparison of CaOH with MTA for direct pulp capping: a PBRN randomized clinical trial. J Dent Res.

[18] Vallés M, Roig M, Duran-Sindreu F, Martínez S, Mercadé M (2015). Color stability of teeth restored with biodentine: a 6-month in vitro study. J Endod.

[19] Marques MS, Wesselink PR, Shemesh H (2015). Outcome of direct pulp capping with mineral trioxide aggregate: a prospective study. J Endod.

[20] Buzdağlı Y, Eyipınar CD, Kacı FN, Tekin A (2022). Effects of hesperidin on anti-inflammatory and antioxidant response in healthy people: a meta-analysis and meta-regression. Int J Environ Health Res.

[21] Tsoupras A (2022). The anti-inflammatory and antithrombotic properties of bioactives from orange, sanguine and clementine juices and from their remaining by-products. Beverages.

[22] Ahmadi A, Shadboorestan A (2016). Oxidative stress and cancer; the role of hesperidin, a citrus natural bioflavonoid, as a cancer chemoprotective agent. Nutr Cancer.

[23] Eliwa ME, Riad D, Hamoda MA, Farouk H (2023). A comparative study of the biological and histological response of eggshell nanoparticles, hesperidin, and calcium hydroxide as direct pulp capping materials: an in vivo study on rat molars. Advanced Dental Journal.

[24] de Cássia Ortiz A, Fideles SO, Reis CH (2022). Therapeutic effects of citrus flavonoids neohesperidin, hesperidin and its aglycone, hesperetin on bone health. Biomolecules.

[25] Suprastiwi E, Putranto AW, Maharti ID (2019). The ability of Biodentine™ of guided tissue remineralization (GTR): analysis using SEM, EDX and TEM. Pesquisa Brasileira em Odontopediatria e Clínica Integrada.

[26] Athanasiadou E, Paschalidou M, Theocharidou A, Kontoudakis N, Arapostathis K, Bakopoulou A (2018). Biological interactions of a calcium silicate based cement (Biodentine™) with stem cells from human exfoliated deciduous teeth. Dent Mater.

[27] Gupta P, Sheikh A, Abourehab MA, Kesharwani P (2022). Amelioration of full-thickness wound using hesperidin loaded dendrimer-based hydrogel bandages. Biosensors (Basel).

